# Impact of an Interdisciplinary Care Program on Health Outcomes in Older Patients with Multimorbidity

**DOI:** 10.3390/jcm14248856

**Published:** 2025-12-15

**Authors:** Pilar Cubo-Romano, Pilar García-de-la-Torre, Carolina Medina-de-Campos, Irene Casado-López, María de-Castro-García, Alejandro Estrada-Santiago, Yolanda Majo-Carbajo, Sara Núñez-Palomares, José Manuel Casas-Rojo

**Affiliations:** 1 Hospital Universitario Infanta Cristina, Parla, 28981 Madrid, Spain; mpilar.garciade@salud.madrid.org (P.G.-d.-l.-T.); carolina.madidac@salud.madrid.org (C.M.-d.-C.); irenemaria.casaso@salud.madrid.org (I.C.-L.); mcastrog@salud.madrid.org (M.d.-C.-G.); alejandro.estrada@salud.madrid.org (A.E.-S.); yolanda.majo@salud.madrid.org (Y.M.-C.); snpalomares@salud.madrid.org (S.N.-P.); jmanuel.casas@salud.madrid.org (J.M.C.-R.); 2 Instituto de Investigación Puerta de Hierro-Segovia de Arana, 28222 Majadahonda, Spain; 3 Facultad de Medicina, Universidad Complutense de Madrid, 28040 Madrid, Spain

**Keywords:** multimorbidity, case manager, chronic care model, comprehensive assessment, individualized care plans

## Abstract

**Background/Objectives**: Evidence on the optimal components and effectiveness of care programs for patients with multimorbidity is limited. This study aimed to evaluate the impact of a structured interdisciplinary program on the incidence of emergency visits, hospitalizations, and avoidable outpatient consultations following an admission or emergency visit. **Methods**: This retrospective observational study included 200 patients enrolled in the Multimorbidity Care Program at Hospital Universitario Infanta Cristina. Event rates were compared during the year before and after program inclusion. Multiple-event survival analysis was performed using the counting process method. **Results**: After program inclusion, patients showed a significant reduction in emergency visits (HR 0.74, 95% CI 0.60–0.92, *p* = 0.006), in conventional hospitalizations (HR 0.54, 95% CI 0.44–0.68, *p* = 0.001), and in avoidable outpatient visits (HR 0.66, 95% CI 0.51–0.86, *p* = 0.005). **Conclusions**: An interdisciplinary care model for patients over 65 with multimorbidity, integrating comprehensive multidimensional assessment, structured patient education, early management of decompensations in a day hospital, and systematic medication review, significantly reduces healthcare utilization. These findings support implementing integrated care programs for complex patients, though multicenter studies and cost-effectiveness analyses are needed to confirm generalizability and sustainability.

## 1. Introduction

The prevalence of chronic diseases in developed societies continues to rise, driven by population aging and changes in lifestyle. In Spain, the population over 65 years is projected to increase from the current 20.4% to 30.5% by 2055, with life expectancy at age 65 reaching 22.7 years for men and 26.3 years for women by 2073 (an increase of 3.3 years) [1]. This demographic shift has led to a rise in multimorbidity, defined as the coexistence of two or more chronic conditions, affecting over 65% of individuals aged 65 and older, and up to 82% of those over 85 [2,3].

Patients with multimorbidity have a higher risk of functional decline, disability, and chronic pain, poorer quality of life, and increased mortality, in addition to complex pharmacological regimens that increase the risk of inappropriate prescriptions, drug interactions, and non-adherence [4,5,6]. Consequently, these patients are the main users of healthcare services, representing more than two-thirds of healthcare expenditure [7].

However, traditional care centered on the management of single diseases is not appropriate for patients with multimorbidity, as the involvement of multiple specialties is associated with fragmented care, duplication of diagnostic tests, difficulties in medication reconciliation, and poorer coordination across levels of care, while often overlooking other factors such as functional status, mental health, or socioeconomic determinants that directly impact health outcomes and resource utilization [8].

It is therefore necessary to identify, within the group of patients with multimorbidity or complex chronic conditions, those at higher risk of adverse events, high service utilization, multiple consultations, and frequent hospital admissions [9], and to propose a model of care that addresses their needs in a holistic and coordinated manner. In this regard, the European JA CHRODIS (Joint Action on Chronic Diseases and Promoting Healthy Ageing across the Life Cycle) framework proposes an Integrated Multimorbidity Care Model focused on delivering patient-centered, coordinated care [10]. This model organizes its recommendations into five key areas: delivery of care through multidisciplinary teams and individualized care plans; decision support based on evidence-based practices; promotion of self-management and shared decision-making; use of information systems to facilitate communication across care levels; and integration of social and community resources to address the patient’s overall needs. Altogether, the model aims to overcome fragmented care and improve health outcomes in people with complex chronic conditions.

Despite these expert proposals and the growing number of studies on multimorbidity, intervention approaches vary widely and their effectiveness remains uncertain [11]. In this context, the present study evaluates the impact of a structured interdisciplinary program, directed at patients over 65 years of age with multimorbidity, on emergency visits, hospitalizations, and avoidable outpatient consultations following an admission or emergency visit. The program includes a comprehensive geriatric assessment, structured health education, proactive follow-up, early management of decompensation in the day hospital, and systematic medication review.

## 2. Materials and Methods

This retrospective observational study included patients enrolled in the Multimorbidity Care Program (MCP) at Hospital Universitario Infanta Cristina, Madrid, between 10 May 2021 and 23 January 2025.

### 2.1. Multimorbidity Care Program

Patients aged over 65 years who presented to the emergency department or were hospitalized, and who met criteria for complex multimorbidity, as defined by Ollero et al. [12], were assessed by an Internal Medicine physician. According to Ollero, chronic diseases are grouped into eight clinical categories based on target organ damage and associated functional impairment, and patients with conditions in two or more of these categories are considered to have complex multimorbidity. This concept arises from the need for a more comprehensive approach to patients with two or more symptomatic chronic diseases, in which it is difficult to determine a primary condition, as they present a comparable degree of complexity, similar potential for destabilization, management difficulties, and interrelations. It could be considered a more targeted approach than the general term “multimorbidity”.

After this medical assessment, those identified as being at high risk of revisits or readmissions were offered voluntary enrollment in the Multimorbidity Care Program (MCP).

The MCP is coordinated by an interdisciplinary team comprising two Internal Medicine specialists, a case-manager nurse, and a social worker. Patients are assessed within 15 days of the index event. Each patient is assigned a reference Internal Medicine physician responsible for supervising care and treatment decisions. The nurse and physician perform a Comprehensive Geriatric Assessment, covering clinical, nutritional, functional, psychological, and social domains. Identified social risks or caregiving difficulties are managed in collaboration with the social worker, who provides information on available community resources.

All pending tests and specialist appointments are reviewed, duplications or low-value visits are canceled, and a systematic medication review is performed, including deprescription and adherence assessment. Structured patient and caregiver education is provided progressively by the nurse, emphasizing early recognition of warning signs. Patients have direct phone access to the nurse during weekdays, and if needed, are seen in the day hospital for evaluation and treatment, avoiding emergency visits.

A hospital-at-home program provides hospital-level care in the patient’s home for patients who meet criteria for hospital admission. For those patients, it is offered as an alternative to conventional hospitalization because it reduces complications such as nosocomial infections, delirium, and functional decline [13,14].

Follow-up visits for all MCP patients occur at three months and every six months thereafter, with monthly nurse calls to reinforce self-care and early detection of warning signs. The case-manager nurse also serves as a liaison with primary care teams, facilitating access to hospital electronic records and communication with the hospital care team.

### 2.2. Study Population and Eligibility

For this study, we included all patients enrolled in the MCP between 10 May 2021 and 23 January 2025. Patients with at least one year of follow-up prior to program enrollment were included, serving as their own controls. Pre-post comparisons of incidence rates of healthcare episodes were performed, covering the year before and the year after enrollment. Follow-up ended at the date of data extraction (23 January 2025) or patient death. However, at the time they were enrolled in the study, not all of them had yet completed the full one-year follow-up period.

### 2.3. Events of Interest and Statistical Analysis

The events of interest were emergency department visits, unplanned hospital admissions, and outpatient consultations. Avoidable outpatient consultations were defined as those substitutable by comprehensive Internal Medicine care, while consultations in specialties such as Oncology, Surgery, and Psychiatry were considered non-avoidable.

A multiple-event survival analysis was conducted using the counting process method, accounting for the timing (days from inclusion date or enrollment in MCP) and frequency of episodes. Cases were defined as censored when the patient did not complete the one-year follow-up period after enrollment in the program or died during that period. Hazard ratios (HRs) with 95% confidence intervals (CIs) were estimated with robust standard errors clustered by patient. Kaplan–Meier curves were plotted considering each event as unique.

Event counts were also compared using incidence rate ratios (IRRs) and 95% CI, estimated through Poisson and negative binomial regression, with the latter providing the best fit according to log-likelihood and Akaike Information Criterion (AIC). A bar chart summarized total episodes pre- and post-enrollment, including only patients with at least one year of post-program follow-up. Statistical analysis was performed with the Stata software package version 18 (StataCorp. 2023. Stata Statistical Software: Release 18. College Station, TX, USA: StataCorp LLC).

## 3. Results

A total of 200 patients aged 65 years and older with complex multimorbidity were included. They were older adults (mean age 82.5 years) with moderate functional dependence and at risk of malnutrition. A high proportion had moderate to severe dementia, reflecting their status as patients with complex multimorbidity. Baseline clinical, functional, nutritional, and cognitive characteristics of the patients are summarized in Table 1.

**Figure 1:**
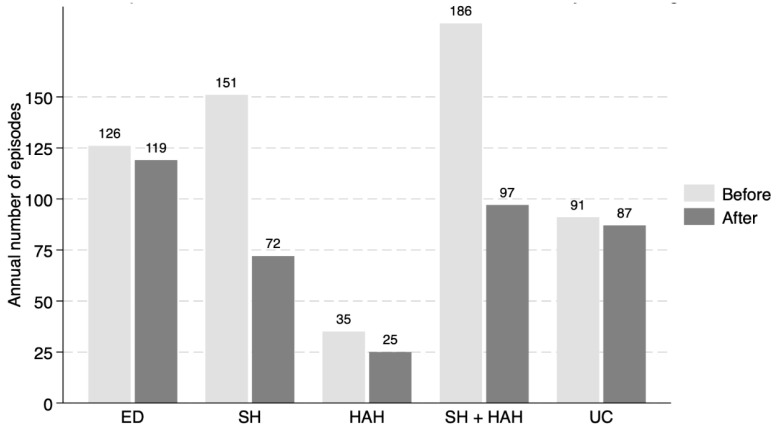
Number of healthcare episodes before and after inclusion in Multimorbidity Care Program. ED: Emergency Department Visits; SH: Standard Hospitalization; HAH: Hospital-at-home care; UC: Unnecessary Medical Consultations.

The absolute number of events was analyzed in 103 patients who had completed one year of follow-up after their inclusion in the program. The number of emergency visits, conventional hospitalizations, hospital-at-home admissions, and avoidable outpatient consultations was lower in the year following program enrollment compared with the year prior (Figure 1).

These findings were further confirmed in pre–post comparisons of incidence rates: The risk of emergency visits decreased (HR 0.74, 95% CI 0.60–0.92, *p* = 0.006), conventional hospitalizations were reduced (HR 0.54, 95% CI 0.44–0.68, *p* = 0.001), and hospital-at-home admissions did not change significantly (HR 0.87, 95% CI 0.54–1.38, *p* = 0.55). The combined outcome of conventional plus hospital-at-home admissions decreased (HR 0.60, 95% CI 0.47–0.75, *p* = 0.001). The risk of hospital outpatient visits also decreased significantly (HR 0.66, 95% CI 0.51–0.86, *p* = 0.005) (Table 2).

**Figure 2:**
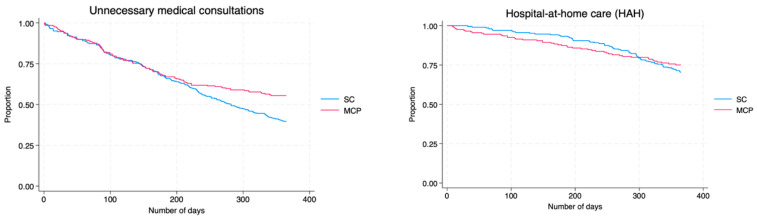

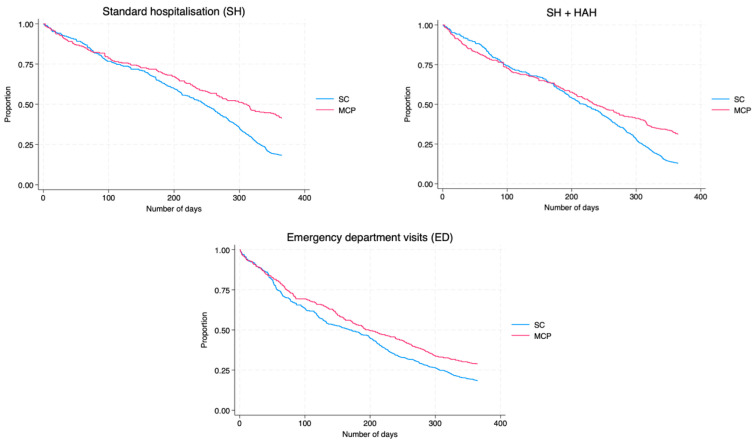
Kaplan–Meier curves of healthcare outcomes incidence after inclusion in the Multimorbidity Care Program. SC: Standard Care; MCP: Multimorbidity Care Program.

Kaplan–Meier curves for each type of event are shown in Figure 2, illustrating the differences in incidence rates before and after program enrollment.

Incidence rate ratios (IRRs) comparing post- versus pre-enrollment periods confirmed these findings. The most pronounced effect was observed for conventional hospitalizations, with a 49% reduction in incidence rate (IRR 0.51, 95% CI 0.41–0.64). Statistically significant reductions were also observed for the combined conventional plus hospital-at-home hospitalizations and for emergency visits. Results are summarized in Table 3.

## 4. Discussion

In this study of patients over 65 years with complex multimorbidity, enrollment in a structured interdisciplinary program was associated with a significant reduction in the risk of emergency visits, conventional hospitalizations, and avoidable outpatient consultations. Pre-post comparisons showed that the risk of emergency visits decreased by 26% (HR 0.74, 95% CI 0.60–0.92), conventional hospitalizations by 46% (HR 0.54, 95% CI 0.44–0.68), and the combined measure of conventional plus hospital-at-home hospitalizations by 40% (HR 0.60, 95% CI 0.47–0.75). In other words, the overall decrease in the risk of hospitalizations was due to a reduction in conventional admissions, with no statistically significant decrease in hospital-at-home admissions (HR 0.87, 95% CI 0.54–1.38, *p* = 0.55). This finding suggests that the program may facilitate a shift from conventional hospitalization to hospital-at-home care, reflecting its potential to modify the mode of patient management and optimize resource use. The risk of hospital outpatient visits also decreased (HR 0.66, 95% CI 0.51–0.86). These findings support the effectiveness of coordinated patient-centered interventions in reducing healthcare utilization among high-risk older adults.

Although the evaluation of programs aimed at patients with multimorbidity is expanding, published results remain heterogeneous and, in many cases, contradictory. This variability is attributable to multiple factors. First, there is no universal consensus regarding terminology or operational definitions in chronic care, which hampers comparative analysis of prevalence and outcomes. Moreover, studies exhibit considerable heterogeneity in both methodological design and the clinical and prognostic characteristics of included populations. Furthermore, many of the interventions analyzed have not demonstrated clear effectiveness. In some cases, available information is insufficient to identify which components of integrated care truly contribute to improved health outcomes. Additionally, direct comparisons between models or intervention strategies are scarce, limiting the ability to determine which approaches are more or less effective in this context [15,16,17].

Consequently, considerable uncertainty remains regarding which model of integrated care is most effective and how such programs should be optimally implemented. Based on this premise, our center’s MCP is founded on the establishment of an interdisciplinary team and the simultaneous implementation of multiple interventions. Unlike multidisciplinary teams—where each professional contributes independently—interdisciplinary teams complement each other’s expertise and work in a coordinated manner toward shared goals, promoting truly patient-centered, integrated care. Previous similar experiences, in which a physician, a nurse, and a social worker conducted follow-up through telephone contact or home visits every six weeks for two years, reported a 2% reduction in hospital readmissions [18].

Within our MCP, a key intervention is the comprehensive geriatric assessment, designed to systematically identify medical, psychological, social, and functional needs, and to develop an integrated, personalized care plan based on these findings. The comprehensive geriatric assessment has been proposed as a key tool for the holistic management of complex patients, enabling the identification of high-risk individuals who may benefit from more efficient integrated programs [19]. Strong evidence indicates that the comprehensive geriatric assessment reduces mortality and disability in hospitalized older adults with acute illnesses [20]. In outpatient older patients with multimorbidity, results are less consistent, although some studies suggest it delays frailty progression and reduces hospitalizations [20,21], while others show limited or inconclusive evidence [22].

Another component of the MCP is structured education for patients and caregivers, delivered by the nurse. Evidence indicates that structured education programs for chronic disease self-management improve several health outcomes, including reductions in fatigue, general malaise, dyspnea, pain, and depressive symptoms, as well as increased medication adherence when interventions focus on self-management. A key aspect is enabling patients and caregivers to recognize early warning signs of disease exacerbations, which is associated with reduced emergency visits and hospital admissions [23,24]. Randomized controlled trials have demonstrated the effectiveness of nurse-led interventions in improving self-management among older adults with multimorbidity [5,25].

The systematic medication review is another core intervention, encompassing reconciliation at hospital discharge and person-centered deprescribing during scheduled consultations. Medication discrepancies during care transitions are common and associated with adverse events, including readmissions. Reconciliation—through compiling comprehensive medication lists, verifying doses, and documenting changes—aims to prevent medication errors. Evidence regarding its impact is mixed: some reviews show no significant reduction in emergency visits or readmissions [26], while others report positive effects [27,28,29]. Person-centered deprescribing is another strategy to address problematic polypharmacy, though results vary; some meta-analyses report statistically significant reductions in hospital readmissions [30], while others show no clinically meaningful improvement [31]. These findings suggest that benefits depend on context, intervention intensity, and interdisciplinary team integration. It is important to note that many of the studies with positive outcomes included in the meta-analysis reporting reduced readmissions [30] incorporated telephone follow-up or home visits during the first 30 days, patient counseling, or both. This approach, also implemented in our program, may have contributed to the positive effects observed in our MCP.

Although evidence for the individual effect of each MCP intervention is limited, measures were selected based on feasibility within the interdisciplinary team framework. Positive outcomes are largely attributed to appropriate patient selection: individuals with multimorbidity and higher risk of emergency visits or readmissions, identified through comprehensive assessment. The holistic perspective of the internist facilitates addressing additional dimensions that affect patient prognosis, such as functional status, sarcopenia, frailty, fall risk, malnutrition, and cognitive or social issues. Furthermore, having the internist responsible for the patient and care process reduces low-value specialty consultations. Finally, proactive nursing calls and patient accessibility to the team enable structured education, strengthen the nurse–patient relationship, and promote early detection of decompensations, thereby preventing unnecessary emergency visits or hospital admissions.

This study has several limitations. First, although the pre–post design allows each patient to serve as their own control, it does not fully eliminate potential confounding. External factors—such as changes in healthcare organization, resource availability, or secular trends—may have influenced the observed outcomes. Second, the study was conducted in a single center with a relatively small sample size, which may limit the generalizability of the findings. Third, not all patients completed a full year of follow-up, which could introduce attrition bias. Fourth, the observational design does not allow us to determine which specific components of the program contributed most to the observed effects; it is possible that the benefit derives from the combined and synergistic action of the interventions rather than any single measure. Fifth, mortality data were not analyzed, and no formal cost-effectiveness evaluation was performed, which would be essential to assess the sustainability and scalability of the program. Finally, the findings need replication in larger, multicenter studies with longer follow-up to confirm their robustness and external validity.

## 5. Conclusions

A care model for patients over 65 years with multimorbidity, based on an interdisciplinary team including a physician, nurse, and social worker, and incorporating a comprehensive multidimensional assessment, structured health education, proactive follow-up, early management of decompensation in the day hospital, and systematic medication review, reduces the risk of emergency visits, hospitalizations, and avoidable outpatient consultations. These findings highlight the potential of structured interdisciplinary programs to improve health outcomes and optimize healthcare resource use in older patients with complex needs. Future multicenter randomized trials and economic evaluations are warranted to confirm scalability and sustainability.

## Figures and Tables

**Table 1:** Baseline clinical, functional, nutritional and cognitive characteristics of the patients.

Variable	Result
Age, mean (SD)	82.5 (7.3)
Female gender, %	50
Barthel index, mean (SD)	78.6 (21.6)
Lawton scale, mean (SD)	4.0 (2.5)
Dawnton scale, mean (SD)	2.6 (1.1)
Chronic Pain, %	24.5
MNA SF index, mean (SD)	10.2 (2.4)
Cognitive status (Pfeiffer scale)	
No cognitive impairment, %	71.9
Mild cognitive impairment, %	15.6
Severe cognitive impairment, %	12.5

The Barthel Index measures basic activities of daily living. The Lawton Scale assesses instrumental activities of daily living. The Downton Scale evaluates the risk of falls. The Mini Nutritional Assessment-Short Form (MNA-SF) assesses nutritional status. The Pfeiffer Scale evaluates cognitive function, categorized as no cognitive impairment, mild, moderate and severe cognitive impairment. Chronic pain was self-reported by the patients.

**Table 2:** Hazard ratios for healthcare outcomes comparing the Multimorbidity Care Program versus Standard Care.

Outcomes	HR (95% CI), Multimorbidity Care Program/Standard Care	*p*
Standard hospitalization	0.54 (0.44–0.68)	<0.001
Hospital-at-home care	0.87 (0.54–1.38)	0.545
SH + HAH	0.60 (0.47–0.75)	<0.001
Emergency department visits	0.74 (0.60–0.92)	0.006
Avoidable outpatient consultations	0.66 (0.51–0.86)	0.002

SH: Standard hospitalization. HAH: Hospital-at-home care. HR: Hazard ratio. CI: Confidence interval.

**Table 3:** Incidence rate ratios for healthcare outcomes comparing the Multimorbidity Care Program versus Standard Care.

Outcomes	Incidence Rate Ratios
Standard hospitalization (SH)	0.51 (0.41–0.64)
Hospital-at-home care (HAH)	0.84 (0.51–1.36)
SH + HAH	0.58 (0.46–0.74)
Emergency department visits	0.77 (0.62–0.95)
Avoidable outpatient consultations	0.81 (0.64–1.03)

SH: Standard hospitalization. HAH: Hospital-at-home care.

## Data Availability

Data available on request due to privacy and legal restrictions.

## Abbreviations

The following abbreviations are used in this manuscript:

ED	Emergency Department visits
SH	Standard Hospitalization
HAH	Hospital-at-home care
UC	Unnecessary Medical Consultations

## References

[1] INEbase/Demografía y Población/Cifras de Población y Censos Demográficos/Proyecciones de Población/Últimos Datos. INE. https://www.ine.es/dyngs/INEbase/es/operacion.htm?c=Estadistica_C&cid=1254736176953&menu=ultiDatos&idp=1254735572981.

[2] Melis R., Marengoni A., Angleman S., Fratiglioni L. (2014). Incidence and predictors of multimorbidity in the elderly: A population-based longitudinal study. PLoS ONE.

[3] Fortin M., Stewart M., Poitras M.E., Almirall J., Maddocks H. (2012). A systematic review of prevalence studies on multimorbidity: Toward a more uniform methodology. Ann. Fam. Med..

[4] Marengoni A., Angleman S., Melis R., Mangialasche F., Karp A., Garmen A., Meinow B., Fratiglioni L. (2011). Aging with multimorbidity: A systematic review of the literature. Ageing Res. Rev..

[5] Yang C., Lee D.T.F., Wang X., Chair S.Y. (2022). Effects of a nurse-led medication self-management intervention on medication adherence and health outcomes in older people with multimorbidity: A randomised controlled trial. Int. J. Nurs. Stud..

[6] Nobili A., Marengoni A., Tettamanti M., Salerno F., Pasina L., Franchi C., Iorio A., Marcucci M., Corrao S., Licata G. (2011). Association between clusters of diseases and polypharmacy in hospitalized elderly patients: Results from the REPOSI study. Eur. J. Intern. Med..

[7] Tinetti M.E., Fried T.R., Boyd C.M. (2012). Designing health care for the most common chronic condition—Multimorbidity. JAMA.

[8] Onder G., Palmer K., Navickas R., Jurevičienė E., Mammarella F., Strandzheva M., Mannucci P., Pecorelli S., Marengoni A. (2015). Time to face the challenge of multimorbidity. A European perspective from the joint action on chronic diseases and promoting healthy ageing across the life cycle (JA-CHRODIS). Eur. J. Intern. Med..

[9] Hempel S., Bolshakova M., Hochman M., Jimenez E., Thompson G., Motala A., Ganz D.A., Gabrielian S., Edwards S., Zenner J. (2023). Caring for high-need patients. BMC Health Serv. Res..

[10] Palmer K., Marengoni A., Forjaz M.J., Jureviciene E., Laatikainen T., Mammarella F., Muth C., Navickas R., Prados-Torres A., Rijken M. (2018). Multimorbidity care model: Recommendations from the consensus meeting of the Joint Action on Chronic Diseases and Promoting Healthy Ageing across the Life Cycle (JA-CHRODIS). Health Policy.

[11] Skou S.T., Mair F.S., Fortin M., Guthrie B., Nunes B.P., Miranda J.J., Boyd C.M., Pati S., Mtenga S., Smith S.M. (2022). Multimorbidity. Nat. Rev. Dis. Primers.

[12] Ollero-Baturone M., Álvarez M., Barón-Franco B., Bernabéu M., Codina A., Fernández A., Garrido E. *Atención al Paciente Pluripatológico. Proceso Asistencial Integrado*, 2nd ed.; Consejería de Salud de Andalucía. https://www.juntadeandalucia.es/export/drupaljda/salud_5af1956d9ec34_pluri.pdf.

[13] Levine D.M., Ouchi K., Blanchfield B., Saenz A., Burke K., Paz M., Diamond K., Pu C.T., Schnipper J.L. (2020). Hospital-Level Care at Home for Acutely Ill Adults: A Randomized Controlled Trial. Ann. Intern. Med..

[14] Leong M.Q., Lim C.W., Lai Y.F. (2021). Comparison of Hospital-at-Home models: A systematic review of reviews. BMJ Open.

[15] Smith S.M., Wallace E., O’Dowd T., Fortin M. (2021). Interventions for improving outcomes in patients with multimorbidity in primary care and community settings. Cochrane Database Syst. Rev..

[16] Rohwer A., Toews I., Uwimana-Nicol J., Nyirenda J.L., Niyibizi J.B., Akiteng A.R., Meerpohl J.J., Bavuma C.M., Kredo T., Young T. (2023). Models of integrated care for multi-morbidity assessed in systematic reviews: A scoping review. BMC Health Serv. Res..

[17] Barajas-Nava L.A., Garduño-Espinosa J., Mireles Dorantes J.M., Medina-Campos R., García-Peña M.C. (2022). Models of comprehensive care for older persons with chronic diseases: A systematic review with a focus on effectiveness. BMJ Open.

[18] Sommers L.S., Marton K.I., Barbaccia J.C., Randolph J. (2000). Physician, nurse, and social worker collaboration in primary care for chronically ill seniors. Arch. Intern. Med..

[19] Hopman P., Schellevis F.G., Rijken M. (2016). Health-related needs of people with multiple chronic diseases: Differences and underlying factors. Qual. Life Res..

[20] Mazya A.L., Garvin P., Ekdahl A.W. (2019). Outpatient comprehensive geriatric assessment: Effects on frailty and mortality in old people with multimorbidity and high health care utilization. Aging Clin. Exp. Res..

[21] Vallejo Maroto I., Cubo Romano P., Mafé Nogueroles M.C., Matesanz-Fernández M., Pérez-Belmonte L.M., Criado I.S., Gómez-Huelgas R., Manglano J.D., Focus Group on Aging of the Spanish Society of Internal Medicine and the Working Group on Polypathology and Advanced Age (2021). Recommendations on the comprehensive, multidimensional assessment of hospitalized elderly people. Position of the Spanish Society of Internal Medicine. Rev. Clin. Esp..

[22] Briggs R., McDonough A., Ellis G., Bennett K., O’Neill D., Robinson D. (2022). Comprehensive Geriatric Assessment for community-dwelling, high-risk, frail, older people. Cochrane Database Syst. Rev..

[23] Cevirme A., Gokcay G. (2020). The impact of an Education-Based Intervention Program (EBIP) on dyspnea and chronic self-care management among chronic obstructive pulmonary disease patients. A randomized controlled study. Saudi Med. J..

[24] Feng C., Wang Y., Li S., Qu Z., Zheng S. (2023). Effect of self-management intervention on prognosis of patients with chronic heart failure: A meta-analysis. Nurs. Open.

[25] Markle-Reid M., Ploeg J., Fraser K.D., Fisher K.A., Bartholomew A., Griffith L.E., Miklavcic J., Gafni A., Thabane L., Upshur R. (2018). Community Program Improves Quality of Life and Self-Management in Older Adults with Diabetes Mellitus and Comorbidity. J. Am. Geriatr. Soc..

[26] Redmond P., Grimes T.C., McDonnell R., Boland F., Hughes C., Fahey T. (2018). Impact of medication reconciliation for improving transitions of care. Cochrane Database Syst. Rev..

[27] Delgado Silveira E., Fernandez-Villalba E.M., García-Mina Freire M., Albiñana Pérez M.S., Casajús Lagranja M.P., Peris Martí J.F. (2015). The impact of Pharmacy Intervention on the treatment of elderly multi-pathological patients. Farm Hosp..

[28] Mekonnen A.B., McLachlan A.J., Brien J.A.E. (2016). Effectiveness of pharmacist-led medication reconciliation programmes on clinical outcomes at hospital transitions: A systematic review and meta-analysis. BMJ Open.

[29] Ciudad-Gutiérrez P., Del Valle-Moreno P., Lora-Escobar S.J., Guisado-Gil A.B., Alfaro-Lara E.R. (2023). Electronic Medication Reconciliation Tools Aimed at Healthcare Professionals to Support Medication Reconciliation: A Systematic Review. J. Med. Syst..

[30] Carollo M., Crisafulli S., Vitturi G., Besco M., Hinek D., Sartorio A., Tanara V., Spadacini G., Selleri M., Zanconato V. (2024). Clinical impact of medication review and deprescribing in older inpatients: A systematic review and meta-analysis. J. Am. Geriatr. Soc..

[31] Cole J.A., Gonçalves-Bradley D.C., Alqahtani M., E Barry H., Cadogan C., Rankin A., Patterson S.M., Kerse N., Cardwell C.R., Ryan C. (2023). Interventions to improve the appropriate use of polypharmacy for older people. Cochrane Database Syst. Rev..

